# Impact of Wheat Resistance Genes on Wheat Curl Mite Fitness and Wheat Streak Mosaic Dynamics Under Single and Mixed Infections

**DOI:** 10.3390/v17071010

**Published:** 2025-07-18

**Authors:** Saurabh Gautam, Kiran R. Gadhave

**Affiliations:** 1 Alliance of Pest Control Districts, Tulare, CA 93274, USA; saurabh@apcd.ca.gov; 2 Texas A&M AgriLife Research and Extension Center, Amarillo, TX 79106, USA; 3 Department of Entomology, Texas A&M University, College Station, TX 77843, USA

**Keywords:** *Wsm1*, *Wsm2*, *Cmc3* and *-4* genes, wheat genetics, virus-host-vector interactions

## Abstract

The wheat curl mite (WCM, *Aceria tosichella* Keifer), a complex of eriophyid mite species, transmits wheat streak mosaic virus (WSMV) and Triticum mosaic virus (TriMV), which in single or mixed infections cause wheat streak mosaic (WSM) disease—a major threat to wheat production across the U.S. Great Plains. Resistant wheat cultivars bearing *Cmc3* and *Cmc4* (targeting WCM), *Wsm1* and *Wsm2* (targeting WSMV), and *Wsm1* (targeting TriMV) are widely used to manage this pest–pathogen complex. However, comprehensive studies investigating how these resistance mechanisms influence both vector biology and virus transmission remain scarce. To address this gap, we evaluated disease development and WCM fitness across nine wheat cultivars with differential resistance profiles under single and mixed infections of WSMV and TriMV. We found strong viral synergy in co-infected plants, with TriMV accumulation markedly enhanced during mixed infections, irrespective of host genotype. Symptom severity and virus titers (both WSMV and TriMV) were highest in the cultivars carrying *Wsm2*, suggesting a potential trade-off in resistance effectiveness under mixed infection pressure. While mite development time (egg to adult) was unaffected by host genotype or infection status, mite fecundity was significantly reduced on infected plants carrying *Wsm1* or *Wsm2*, but not on those with *Cmc3* and *Cmc4*. Notably, virus accumulation in mites was reduced on the cultivars with *Cmc3* and *Cmc4*, correlating with virus titers in the host tissues. Our findings highlight the complex interplay between host resistance, virus dynamics, and vector performance. Cultivars harboring *Cmc3* and *Cmc4* may offer robust field-level protection by simultaneously suppressing mite reproduction and limiting virus accumulation in both plant and vector.

## 1. Introduction

Mite-vectored viral pathogens—wheat streak mosaic virus (WSMV, genus *Tritimovirus*, family *Potyviridae*), Triticum mosaic virus (TriMV, genus *Poacevirus*, family *Potyviridae*), and High Plains wheat mosaic virus (HPWMoV, genus *Emaravirus*, family, *Fimoviridae*)—are amongst the most economically devastating pest concerns to wheat (*Triticum aestivum* L.) production in the Great Plains of the United States [1,2,3,4]. These viruses, either singly or in mixed infections, cause wheat streak mosaic (WSM) disease. WSMV was first reported in Nebraska in 1954 [2], HPWMoV was first identified in 1993 on wheat and corn (maize, *Zea mays*) in the Great Plains region [5], whereas TriMV was first reported in Kansas in 2008, followed by several other states in the Great Plains [4,6]. Previously, most WSM epidemics have been attributed to WSMV; however, a recent field survey conducted throughout the region revealed that the mixed infection of WSMV and TriMV is much more prevalent than previously reported [6,7,8]. Previous studies have shown that TriMV infections appear to be dependent on WSMV. Also, WSMV and TriMV exhibit synergism when they co-infect wheat through increased titers of both viruses, greater symptom expression, and increased yield loss [9,10]. Although, the degree of synergism can vary depending on the wheat cultivars [10].

Both WSMV and TriMV are transmitted by the wheat curl mite (WCM) complex comprising at least 29 genetic lineages (*Aceria tosichella* Keifer) [2,11]. Wheat curl mites can infest more than 90 grass species, such as wheat, oats, barley, pearl millet, corn, rye, and other cultivated (pasture) and uncultivated grasses [12]. WCM are microscopic arthropods about 0.2 mm in length and have very short mouthparts (chelicerae), which limits their ability to feed only on epidermal tissues. In wheat, under high population pressure, intensive WCM feeding on leaf epidermal tissues, especially thin-walled bulliform cells within the whorl of a developing leaf, prevents the unfurling of affected leaves and results in the characteristic leaf curling associated with WCM-infested wheat plants [13]. At 25 °C (77 °F), WCM completes their life cycle (an egg, two instars of nymph, and an adult) in an average of 7 days [2]. In the Great Plains, two genotypes of WCM, Type 1 and Type 2 (globally, MT-1 and MT-8), are reported to be dominant species in infested wheat fields [14]. Both genotypes differ in virus transmission characteristics; the Type 2 mite is reported to be an efficient vector for both WSMV and TriMV [15,16]. Under field conditions, virus transmission mainly occurs when viruliferous mites are transported by the wind from infected to non-infected plants. Both nymphs and adults can transmit WSMV, but the acquisition of the virus from diseased plants is restricted to nymphs [2]. WSMV transmission appears persistent and circulative, as virus-like particles and inclusion bodies have been found in the digestive tracts of viruliferous mites [17,18,19]. Nymphs acquire the virus while feeding on infected wheat, and WCM remains viruliferous through molting [17,20].

The broad host range of both viruses and mites and the concealed feeding and growth of WCM make removing alternate host plants and applying pesticides to control them ineffective. Therefore, the management of WCM and the disease complex has conventionally focused on an integrated pest management (IPM) approach that combines removing the “green bridge” plants that allow the complex to overwinter or survive over summer [21,22] with wheat cultivars tolerant or resistant to the mite or viruses [23,24]. There are no wheat cultivars introgressed with genetic resistance to all pathogens in the WSM complex, but several are resistant to WSMV and/or WCM [24,25,26,27]. Several wheat cultivars that are commercially available to Great Plains growers carry only one of the following identified sources of genetic resistance: *Wsm1*, *Wsm2*, *Cmc1*, *Cmc3*, or *Cmc4*. Both *Wsm1* and *Wsm2* provide resistance against WSMV, while *Wsm1* can also provide resistance against TriMV [24]. Both genes are temperature sensitive and are reported to provide more effective resistance at 18 °C than at 24 °C or above [28,29,30,31,32,33]. *Curl Mite Colonization* (CMC) loci confer resistance to WCM by inhibiting mite reproduction, thereby reducing the spread of the viruses [34]. The *Cmc* gene-mediated resistance is reported to interact differently with different WCM populations [35].

Given the increasing evidence of mixed infections of WSMV and TriMV in wheat fields and in some instances TriMV over WSMV, a comprehensive study evaluating the different forms of single gene resistances in wheat against WSMV, TriMV, and WCM is imperative to understand the ever-changing pathogen dynamics and transmission biology. This information will aid wheat breeders to select for genes and traits that provide better and more complete protection against various components of this complex threat. It is also expected to help growers select cultivars or tailor strategies based on the prevalence of pathogen/mite pressure each year. Therefore, in the current study, we conducted a series of bioassays to understand how the single vs. mixed infection of both viruses impacts the first onset of symptoms (i.e., incubation), disease severity, and virus accumulation in a panel of nine differentially resistant wheat cultivars (Table 1). Mixed-virus infections in host plants can affect vector fitness and virus acquisition differently than single-virus infections, thereby profoundly impacting disease epidemics. Therefore, we also studied how the single vs. mixed infection of these viruses in the cultivar panel impacts WCM fitness (developmental mite and fecundity) and subsequent TriMV and/or WSMV acquisition from singly or mixed infected plants.

## 2. Materials and Methods

### 2.1. Wheat Varieties, Virus Isolate, and Mite Population

This study used one susceptible cultivar and nine resistant wheat cultivars carrying the *Wsm1*, *Wsm2*, *Cmc3*, and/or *Cmc4* genes (Table 1). In March 2022, leaf bits from five field-planted TAM 304 plants (susceptible wheat variety) showing characteristic WSM disease symptoms (light green streaks and mosaic pattern) were collected and tested for WSMV, TriMV, and HPWMoV infection via qRT-PCR analysis [8,10]. All collected WSM symptomatic plant tissues from the field were mixed infected with WSMV and TriMV, while none had HPWMoV. To separate TriMV and WSMV, 100 mg of mixed infected leaf tissues were mechanically inoculated onto ten seedlings (1–2 weeks old) of corn (*Zea mays* L.) and barley (*Hordeum vulgare* L.) of unknown cultivars. The seedlings were transferred and maintained in a growth chamber (20 ± 1 °C, 14 h L:10 h D). Three weeks post inoculation, total RNA from 25 mg of the youngest leaf tissue was extracted, and the presence of WSMV and TriMV was tested via qRT-PCR. Singly (WSMV: corn or TriMV: barley) and mixed infected (WSMV and TriMV: wheat) plants were transferred and maintained in a separate growth chamber at 20 ± 1 °C, 14 h L:10 h D. After one week of acclimatization, leaf pieces cut to 2–3 cm length infested with different stages of WCM were transferred to singly and mixed infected plants using forceps. After one week of feeding, 2–3 cm singly or mixed infected leaf bits of corn, barley, or wheat infested with mites were collected and transferred to five 2–3-week-old TAM 304 plants maintained in a growth chamber under the conditions specified above. After three weeks of infestation, all symptomatic plants were tested for the presence of TriMV and/or WSMV using qRT-PCR. As expected, newly inoculated TAM 304 plants infested with mites from corn, barley, and TAM 304 plants were infected only with WSMV, TriMV, and both viruses, respectively. These singly or mixed infected TAM 304 plants were used as a source of inoculum for WSMV and/or TriMV in the experiments described below. Throughout the study, singly or mixed infection TAM 304 plants were maintained in separate growth chambers, with repeated inoculations of TAM 304 seedlings with viruliferous mites as needed. Inoculum sources of TriMV and/or WSMV were routinely tested to rule out cross-contamination between the two viruses.

### 2.2. Mites

Wheat curl mites (WCM, *Aceria tosichella* Keifer) Type 2 used in the present study were first collected from the Bushland Research Farm, Bushland, TX, as described by Dhakal et al. [35]. Throughout the study, viruliferous and non-viruliferous mite populations were maintained on infected (WSMV and/or TriMV) or non-infected TAM 304 plants in separate growth chambers.

### 2.3. Effect of Single vs. Mixed Infection of WSMV and TriMV on Incubation Period, Symptom Severity, Disease Incidence, and Virus Accumulation in Wheat Cultivars with Varying Genetics

This experiment was conducted in a greenhouse to simulate warm early-fall conditions, which are typically associated with an increased incidence of wheat streak mosaic (WSM) even in otherwise-resistant cultivars. During the experiment, average temperatures inside the insect-proof cages ranged from 20 ± 1.15 °C at night to 20 ± 2.67 °C during the day. Wheat seedlings from the cultivars listed in Table 1 were planted individually in 10 cm plastic pots (4 cm depth). Two to three weeks after germination, seedlings were infested with either viruliferous mites (carrying WSMV and/or TriMV) or non-viruliferous mites. The experimental design included four treatments: single infection with WSMV, single infection with TriMV, mixed infection with both viruses, and a non-infected control. Each infested plant in an individual pot was treated as a replicate (i.e., an experimental unit). For each cultivar and treatment, 14 replicate plants were established and distributed equally between two insect-proof cages (7 plants per cage; *n* = 14). The cages were used to prevent cross-contamination by mites, viruses, or other opportunistic pests and were placed on greenhouse benches for the duration of the experiment.

After one week of infestation, plants were monitored daily at 10 AM for WSM disease symptoms. After three weeks of infestation, disease severity was rated based on a scale of 1 to 5 (1 = no chlorosis on leaf; 2 = one to few chlorotic streaks; 3 = <25% of leaf areas with chlorosis; 4 = 25–50% of leaf areas with chlorosis; 5 = whole leaf areas with chlorosis) [35]. After disease severity scoring, leaf bits from the most symptomatic parts were collected for WSMV and TriMV quantitation. Approximately 10 mg of symptomatic leaf tissue was freeze-dried overnight using a Labconco FreeZone 2.5 L benchtop freeze dryer (Labconco Corporation, Kansas City, MO, USA) prior to RNA extraction. Frozen tissues were ground using a Geno/Grinder^®^ (SPEX SamplePrep, LLC, Metuchen, NJ, USA), and total RNA was extracted using TRIzol^®^ reagent (Thermo Fisher Scientific, Waltham, MA, USA). Absolute copies of TriMV and WSMV in 10 ng of total extracted RNA were estimated using serial dilutions of known standards using the procedure described earlier by Gautam et al., (2023) [36]. The experiments were conducted under greenhouse conditions intended not to fully replicate but to approximate the warm field conditions typically experienced in the Texas High Plains during late fall. Winter wheat planted in late fall is more susceptible to early infections of WSM. In the High Plains of Texas, winter wheat is planted from mid-September to October and harvested in the summer, starting in late May. Infections in late spring, due to increasing temperatures, may not cause yield losses.

### 2.4. Effect of Single vs. Mixed Infection of WSMV and TriMV in Wheat Cultivars on Wheat Curl Mite Developmental Time, Fecundity, and Virus Acquisition

Due to the WCM’s microscopic size and concealed feeding, the daily monitoring of mite development on whole plants turned out to be cumbersome and inaccurate in some instances after our first few attempts. Therefore, we used 2–3 cm leaf bits from non-infected or infected (singly or mixed) wheat plants across different cultivars to evaluate the mite egg to adult developmental times. For mite biology studies, infected plants were obtained via the mechanical inoculation of 2–3-week-old seedlings from different cultivars. Using a fine paintbrush with a single hair, an adult WCM female (identified based on size and yellowish appearance) was picked from a non-infected colony under a dissecting microscope and transferred on a leaf bit in a 3 cm Petri plate lined with moist Whatman filter paper. The adult female was removed after the 24 h oviposition period, and the leaf bit was carefully examined under the microscope for the presence of eggs. All but one egg on each leaf bit were removed, and leaf bits without eggs were disregarded from the experiment. Single egg-bearing Petri plates were maintained in a growth chamber at the conditions specified earlier. Petri plates were resupplied with new leaf bits every alternate day, and old dried ones were removed. From initial trial experiments, we learnt that mites take at least six days to reach adulthood and that daily exposure to microscope light significantly increases mite mortality in Petri plates, possibly due to desiccation. To avoid this, leaf bits were observed every day from the 5th day onwards, until the first progeny (F1) adult deposited the first egg or until the observed F1 progeny died. A Petri plate with a leaf bit constituted an experimental unit for the treatments. For each treatment, the experiment consisted of 15 replications (experimental units). Due to mite mortality, final treatment replications ranged between 8 and 11. Each cultivar had four treatments: singly infected plants (WSMV or TriMV), mixed infected plants (TriMV and WSMV), and non-infected mock-inoculated plants.

For the fecundity experiment, four treatment groups were established for each wheat cultivar: plants singly infected with WSMV or TriMV, plants with mixed infection (WSMV + TriMV), and mock-inoculated non-infected controls. Infections were established via mechanical inoculation. To standardize the age of the mites, leaf bits containing mite eggs were placed onto replicate plants for each treatment group. After one week of infestation, seedlings were examined under a dissecting microscope to identify WCM females. These females, having developed on specific treatments, were transferred to fresh leaf bits from the same treatment group. Each leaf bit, containing a single female, was placed in a 3 cm Petri dish lined with moist filter paper to constitute one replicate. Females were transferred to new leaf bits every other day, and the number of eggs laid on the previous leaf bit was recorded. Fecundity was monitored for 12 days. The experiment began with 15 replicates per treatment. However, due to mite mortality associated with frequent handling and transfer, the final number of replicates ranged from 7 to 11 across treatments.

To assess WSMV and TriMV accumulation in mites developing on different wheat cultivars, 2–3-week-old seedlings from the cultivar panel described in Table 1 were infested with either viruliferous (WSMV and/or TriMV) or non-viruliferous wheat curl mites. This resulted in four treatment groups: single infections with WSMV or TriMV, mixed infection with both viruses, and a non-infected control group inoculated with non-viruliferous mites. Following infestation, four plants of the same cultivar and treatment group were placed in insect-proof cages within a greenhouse. During the experiment, average temperatures inside the cages were maintained at 20 ± 1.15 °C (night) and 20 ± 2.67 °C (day). After four weeks, mite-infested leaf bits were collected from each treatment group. Using a fine brush under a dissecting microscope, ten mites (nymphs and/or adults) were isolated from infected plant tissues and pooled into 1.5 mL centrifuge tubes containing 20 µL of TRIzol reagent. Each tube, containing a pooled sample of ten mites, constituted one replicate. The experiment was conducted with ten replicates per experiment (*n* = 10).

### 2.5. Data Analysis

Data were analyzed in R version 3.6.0 [36]. Data for WSMV and/or TriMV accumulation in singly and mixed infected plants, viruliferous mites, and WCM fecundity were analyzed with the ‘Lme4’ package following a linear mixed model procedure [37]. Virus accumulation and fecundity data were log-transformed before analysis. During analysis, replications were considered random, and treatments were considered fixed effects. ANOVA was run on the model using the function ‘Anova’ in the ‘car’ package [38]. Tukey’s post hoc tests compared means for virus accumulation with the ‘contrast’ and ‘lsmeans’ functions from the ‘lsmeans’ package [39]. The nonparametric Kruskal–Wallis test analyzed the median development time from egg to adult and the time required for symptom development post inoculation in different cultivars. Statistical differences were considered significant at *p* < 0.05. Differences in the WSM disease incidence in different wheat cultivars upon inoculation by mites with WSMV and/or TriMV were evaluated using a binary distribution (diseased vs. non-symptomatic). The model was fitted using the ‘glmer’ function by setting the family argument as ‘binomial’. Treatments were considered fixed effects, and the experiment replications were considered random effects. An analysis of variance (ANOVA) was run on the model using the function ‘Anova’ in the ‘car’ package [38]. A post hoc test was performed with the ‘glht’ function using Tukey’s adjustments for pairwise comparisons in the ‘multcomp’ package [40]. Correlation analyses were conducted to examine the relationships between (1) virus accumulation in plants and disease severity, (2) virus accumulation in plants and in mites feeding on them, using the ‘cor()’ function in R. For the first analysis, due to the non-continuous and non-normally distributed nature of the data, Spearman’s rank correlation was applied. For the second analysis, which involved continuous and normally distributed data, Pearson’s correlation was used.

## 3. Results

### 3.1. Effect of Single vs. Mixed Infection of WSMV and TriMV on Incubation Period, Symptom Severity, and Virus Accumulation in Wheat Cultivars with Varying Genetics

**Figure 1:**
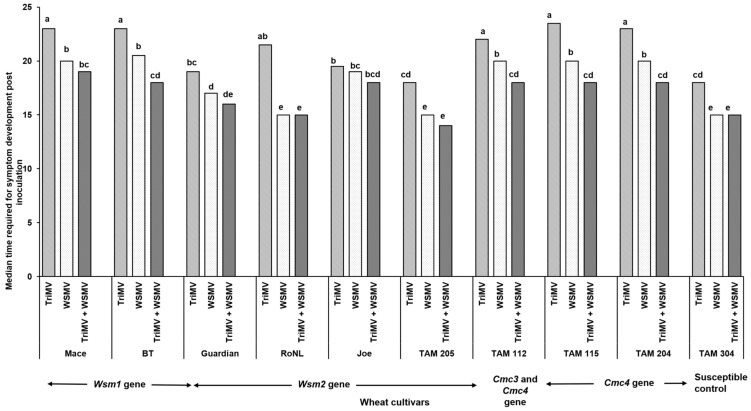
Bars show the median time required for the wheat streak mosaic-specific symptoms to appear in plants belonging to various cultivars after infestation with viruliferous mites. Wheat curl mites developing on (TriMV and/or WSWV)-infected wheat were released on wheat cultivars with varying genetics (carrying *Wsm1*, *Wsm2*, *Cmc3*, or *Cmc3* and *Cmc4* resistant genes). Plants were observed every 24 h post infestation for any disease-associated symptoms (chlorosis and chlorotic streaks). The letters on the error bars indicate significant differences between means at *α* = 0.05.

**Figure 2:**
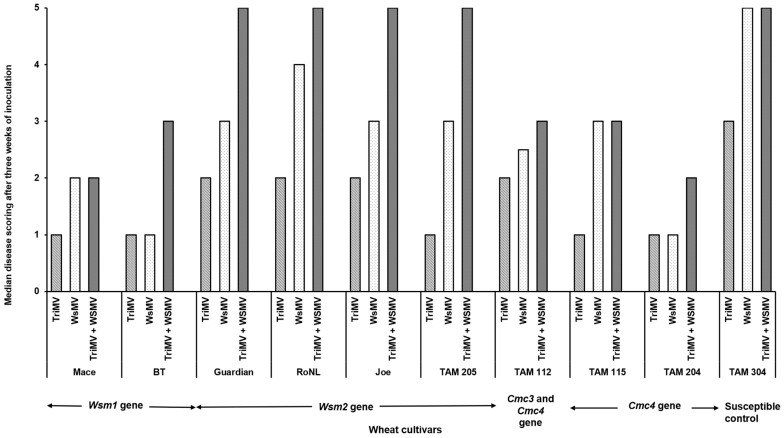
Wheat streak mosaic disease ratings in wheat plants (susceptible and resistant) infested with mites carrying TriMV and/or WSMV. After three weeks of infestation, disease severity is rated based on a scale of 1 to 5 (1 = no chlorosis on leaf; 2 = one to few chlorotic streaks; 3 = <25% of leaf areas with chlorosis; 4 = 25–50% of leaf areas with chlorosis; 5 = whole leaf areas with chlorosis.

The percentage of infection did not differ significantly between the singly or mixed infected plants from different cultivars (*χ*^2^ (29, *N* = 600) = 35.55, *p* = 0.18) (Table 2). However, the post inoculation median time required for symptom development (i.e., incubation period) differed significantly between cultivars and depended on cultivar genetics and infection status (singly or mixed) (*χ*^2^ (29, *N* = 416) = 342.21, *p* < 0.01). Except for Joe (*Wsm2* gene), for every cultivar, the plants infested with mites carrying only TriMV took the longest to show symptoms compared with the ones infected with WSMV or WSMV and TriMV (Figure 1). Consistent with this, the plants infected only with TriMV had the lowest median symptom severity across all cultivars (Figure 2). Except for Mace (*Wsm1*), TAM 115 (*Cmc4*), and the susceptible control, across all cultivars, the mixed infected plants had severe disease phenotypes compared with singly infected plants, highlighting the synergistic effect of mixed infections of WSMV and TriMV. In mixed infected plants, a higher disease severity was observed in the cultivars carrying the *Wsm2* gene than the ones carrying the *Wsm1* or *Cmc3* and/or *Cmc4* genes.

**Figure 3:**
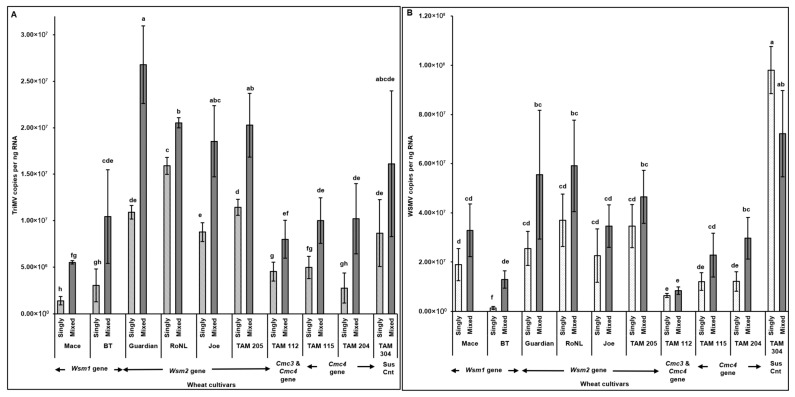
Virus accumulation in wheat plants with varying genetic backgrounds (susceptible and resistant) infested with mites carrying TriMV and/or WSMV. Bars with standard errors represent the average number of TriMV (**A**) and WSMV (**B**) CP gene copies accumulated in singly (TriMV or WSMV) or mixed (TriMV and WSMV) infected susceptible and resistant wheat plants (carrying *Wsm1*, *Wsm2*, *Cmc3*, or *Cmc3* and *Cmc4* resistant genes). Coat protein (CP) gene copy numbers were estimated by qRT-PCR, followed by absolute quantitation using plasmids containing CP gene inserts as standards. The letters on the error bars indicate significant differences between means at *α* = 0.05. The *Y*-axis has a logarithmic scale.

The observations of disease severity were generally consistent with virus accumulation (Figure 3). Spearman’s correlation revealed a significant positive correlation between virus accumulation in plants across different cultivars and disease severity (*rs* (278) = 0.83, *p* < 0.001). High TriMV (*F* = (19, 260) = 4.93; *p* < 0.01) and/or WSMV (*F* = (19, 260) = 12.31; *p* < 0.01) accumulation in mixed infected plants led to the severe disease phenotype in mixed infections. TriMV accumulation was consistently significantly higher in mixed infected plants than in single infections (Figure 3A). High variability was observed in WSMV accumulation between singly and mixed infected plants, and WSMV was always numerically higher in mixed infected resistant plants. However, for only two resistant cultivars (BT: *Wsm1* gene and TAM 204: *Cmc4* gene), WSMV accumulation was significantly higher in mixed infections than in single infections (Figure 3B).

### 3.2. Effect of Single vs. Mixed Infection of WSMV and TriMV on Developmental Time, Fecundity, and Virus Accumulation in Mites

**Figure 4:**
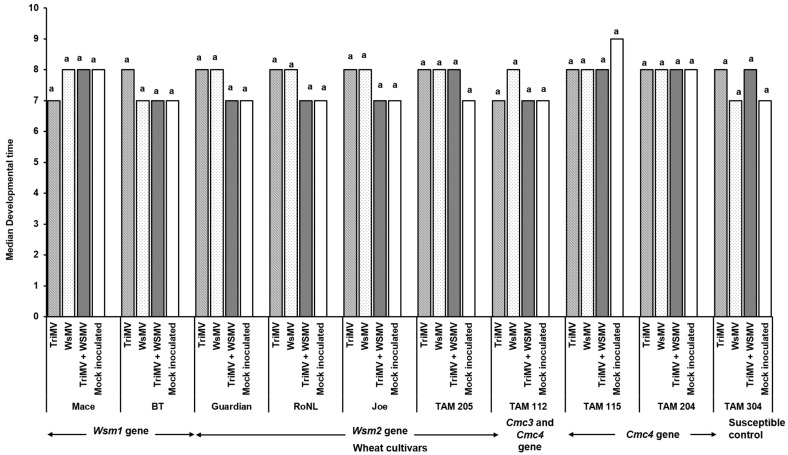
Wheat curl mite developmental time on non-infected and infected (TriMV and/or WSMV) wheat plants belonging to various wheat cultivars with varying genetic backgrounds. Bars show median mite developmental time (egg to adult) on non-infected or infected (singly, TriMV or WSMV, or mixed, TriMV and WSMV) susceptible and resistant wheat plants (carrying *Wsm1*, *Wsm2*, *Cmc3*, or *Cmc3* and *Cmc4* resistant genes). Developmental time was estimated according to wheat leaf sheath maintained in the Petri plate lined with moist filter paper. The letters on the bars indicate significant differences between the median at *α* = 0.05.

**Figure 5:**
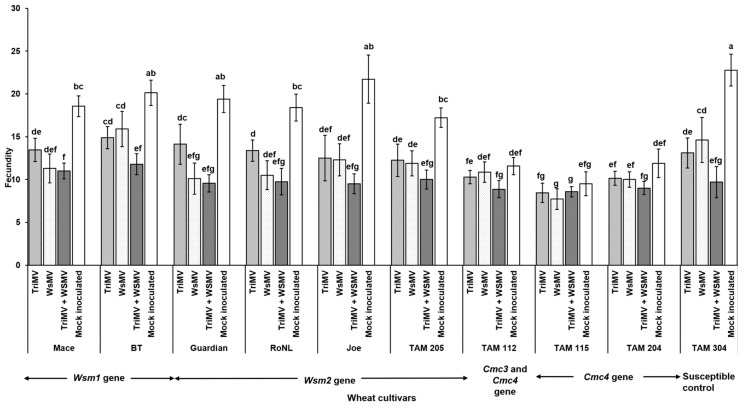
Wheat curl mite fecundity on non-infected and infected (TriMV and/or WSMV) wheat plants belonging to various wheat cultivars with varying genetic backgrounds. Bars with standard errors show fecundity of mites on non-infected or infected (singly, TriMV or WSMV, or mixed, TriMV and WSMV) susceptible and resistant wheat plants (carrying *Wsm1*, *Wsm2*, *Cmc3*, or *Cmc3* and *Cmc4* resistant genes). Fecundity was estimated according to wheat leaf sheath maintained in the Petri plate lined with moist filter paper. Fecundity was recorded for 12 days. The different letters on the error bars indicate significant differences between means at *α* = 0.05.

Mite egg to adult median developmental time did not differ between non-infected and WSMV- and/or TriMV-infected plants across any of the cultivars (*χ*^2^ = 31.7, df = 36, *p* = 0.87). It ranged between 7 and 9 days across the treatments (Figure 4). However, mite fecundity differed significantly across the cultivars and was mostly driven by host genetics (*F* (39, 332) = 7.23; *p* < 0.01) (Figure 5). More specifically, among the treatments belonging to the same cultivar carrying the *Wsm1* or *Wsm2* genes and in the TAM 304 susceptible control, mite fecundity was significantly reduced in infected plants regardless of their infection status (single or mixed) compared with the non-infected controls of each cultivar (Figure 5). While mixed infected plants appear to have lower fecundity than single infections in *Wsm1*- and *Wsm2*-carrying cultivars and TAM 304, the only significant difference was observed in BT, in that mixed infected plants had significantly lower fecundity than TriMV- or WSMV-infected plants. On the contrary, in the cultivars carrying the *Cmc3* and *Cmc4* genes, no significant difference was observed between infected plants (single or mixed) and their respective non-infected controls.

**Figure 6:**
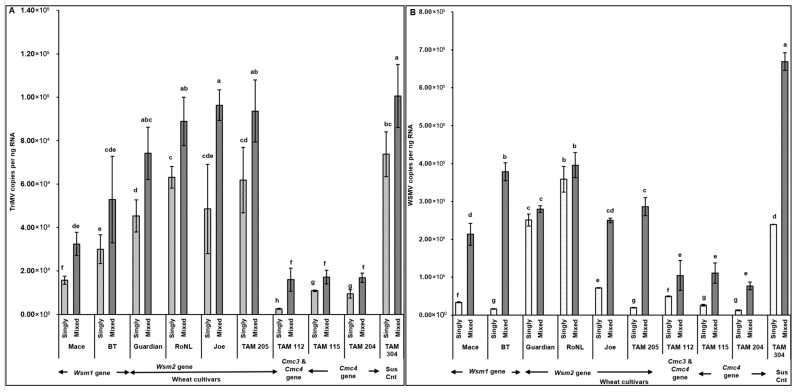
Virus accumulation in mites feeding on wheat plants with varying genetics (susceptible and resistant) infected with TriMV and/or WSMV. Bars with standard errors represent the average number of TriMV (**A**) and WSMV (**B**) CP gene copies accumulated in mites feeding on singly (TriMV or WSMV) or mixed (TriMV and WSMV) infected susceptible and resistant wheat plants (carrying *Wsm1*, *Wsm2*, *Cmc3*, or *Cmc3* and *Cmc4* resistant genes). Coat protein (CP) gene copy numbers were estimated by qRT-PCR, followed by absolute quantitation using plasmids containing CP gene inserts as standards. The letters on the error bars indicate significant differences between means at *α* = 0.05. The *Y*-axis has a logarithmic scale.

TriMV (*F* (19, 190) = 4.68; *p* < 0.01) and WSMV (*F* (19, 190) = 14.56; *p* < 0.01) accumulation in mites feeding on singly (TriMV or WSMV) or mixed (TriMV and WSMV) infected plants differed depending on the wheat cultivar’s genetics. Interestingly, WSMV or TriMV accumulation in mites feeding on mixed infected plants carrying the *Cmc3* and/or -*4* genes was significantly reduced compared with the cultivars carrying *Wsm2* genes. Pearson’s correlation revealed a significant positive correlation between virus accumulation in plants across different cultivars and in the mites feeding on them (*r* (278) = 0.77, *p* < 0.001). Across all treatments, WSMV and TriMV accumulation in mites (i.e., sink) followed the same patterns as the plants from which the mites acquired the virus (i.e., source), suggesting a strong source–sink relationship (Figure 3A,B and Figure 6A,B).

## 4. Discussion

Wheat curl mite-transmitted wheat streak mosaic (WSM) causes substantial economic losses in small grain crops, particularly across the Great Plains of the United States. Historically, wheat streak mosaic virus (WSMV) has been considered the predominant causal agent within this disease complex. However, recent studies suggest that Triticum mosaic virus (TriMV) is also a key driver of WSM symptomology and is most often detected in mixed infections [6,7]. In our study, we found that WSMV and TriMV act synergistically to intensify disease severity compared with single infections, regardless of wheat cultivar genotype (*Wsm1*, *Wsm2*, *Cmc3*, or *Cmc4*). This synergism appears to be primarily driven by TriMV, as its accumulation in co-infected plants was substantially higher than in singly infected ones. In mixed infections, both symptom severity and virus accumulation (WSMV and TriMV) were notably greater in the cultivars carrying the *Wsm2* gene than in those carrying *Wsm1*, *Cmc3*, or *Cmc4*. Although wheat curl mite (WCM) development from egg to adult proceeded at a comparable rate across all cultivars, irrespective of infection status or genetic background, mite fecundity was significantly influenced by both factors. Fecundity was markedly reduced in infected plants carrying *Wsm1* or *Wsm2* compared with their non-infected counterparts, a trend not observed in cultivars with *Cmc3* and/or *Cmc4*. Similarly, virus accumulation in WCM was modulated by host plant genotype and infection status. WSMV and/or TriMV titers were reduced in mites feeding on cultivars with *Cmc3* and/or *Cmc4* resistance, and virus accumulation in mites was density-dependent—higher titers in mites corresponded to higher titers in the host plants. This study unravels the dynamics of virus–vector–host interactions in differentially resistant wheat cultivars and highlights the downstream consequences for vector fitness. While previous investigations of WSM pathogen dynamics have been conducted in isolation under controlled conditions with limited host diversity, this study’s comparative analysis of the mite-mediated transmission of TriMV and/or WSMV across a genetically diverse cultivar panel—and the implications of single versus mixed infections on mite biology—represents a novel and significant contribution.

Tatineni and co-workers found that at 20–26 °C, the symptoms induced by TriMV on two susceptible and one resistant cultivars were milder than those induced by WSMV [10]. Furthermore, WSM symptom development was delayed in a single infection of TriMV compared with WSMV alone or a mixed infection, and the mixed infection of both viruses produced a severe phenotype in susceptible plants compared with the resistant one. Consistent with Tatineni et al., we observed that the median time required for symptom development was longer for a single infection of TriMV than WSMV or mixed infection [10]. Furthermore, except for the *Wsm2* cultivar, the *Wsm1* and *Cmc3* and/or *4* cultivars had lower disease severity ratings than susceptible plants. The increased severity of mixed infected *Wsm2* cultivars was possibly due to two primary reasons; first, *Wsm2* offers protection only against WSMV unlike *Wsm1*, which offers protection against both WSMV and TriMV. These plants therefore were unable to withstand the increased accumulation of TriMV in mixed infected plants, producing a more severe disease phenotype. Secondly, *Wsm2*-derived resistance was reported to be more vulnerable to breakdown at higher temperatures than *Wsm1* [32,41]. Since the temperatures in our study likely reached 23 °C on multiple occasions, it is plausible that *Wsm2*-derived resistance was disrupted which led to a more severe phenotype compared with others.

The *Cmc3* and/or *Cmc4* genes are likely to have reduced or prevented mite feeding as reported by Nachappa et al., (2021) leading to reduced WSMV and/or TriMV inoculation and accumulation in all *Cmc* cultivars (TAM 112, TAM 115, and TAM 204) [24]. However, this did not result in a significantly lower percentage of infection in cultivars carrying these genes. These cultivars were also reported to carry mild to moderate resistance or tolerance to WSMV [29] further contributing to longer disease development, lower disease severity, and reduced TriMV and/or WSMV accumulation in plants as well as mites. The synergy between WSMV and TriMV in producing a severe disease phenotype was also observed by Tatineni et al. (2010) [10]. Such synergism was reported to be asymmetrical and depending on the order of plant infection by WSMV and TriMV [42]. In TriMV-infected wheat, WSMV showed accelerated long-distance movement and increased accumulation, whereas, TriMV showed delayed systemic infection in WSMV-infected wheat, with fewer genomic RNA copies in the early stages of infection, which then increased in the later stages of infection. Since the mode and mechanisms of WSMV and TriMV transmission by WCM remain poorly understood, it is not clear whether the simultaneous inoculation of both viruses by mites impacts the incubation time and TriMV and/or WSMV accumulation in the cultivars used in the current study. Further work is needed to establish this with mite transmission and cultivars with varying genetic backgrounds.

The WCM egg to adult developmental time did not differ between the cultivars and ranged 7–9 days, which is consistent with previous findings [43], where it was reported to be 6–11 days. The effect of virus infection on WCM population growth on wheat cultivars is context-specific and varies between studies [44,45]. Currently, there are no prior studies assessing the impact of WSM on mite fecundity. Perhaps the closest metric we can use is mite population density. Murugan et al. reported that WSMV infection in moderately resistant cultivars significantly increased mite populations compared with mock-inoculated plants [45]. However, this was not the case with susceptible plants. Siriwetwiwat demonstrated that WSMV infection significantly increased their reproductive rate on wheat *cv*. Alliance [44]. Contrary to both studies, we found reduced mite fecundity on both resistant and susceptible plants infected with WSMV. Taken together, it appears that the reproductive response of mites on wheat is a complex interplay of many factors, including virus infection, wheat cultivar, and environmental and non-environmental factors. We did not find any prior studies comparing the impacts of mixed infection on WCM development and fecundity. Previous transmission studies using single-mite transfers from infected to non-infected plants have reported that, compared with single infection, co-infection reduced and increased WSMV and TriMV transmission, respectively [16]. Contrary to these findings, we observed higher WSMV and TriMV accumulation in mites feeding on mixed infected plants over singly infected plants. A similar transmission rate study will offer more insights into the transmission biology of mites.

The overall genetic makeup of a cultivar—its genetic background—can influence not only the expression and efficacy of resistance genes but also factors such as virus movement and the selection pressure exerted on virus populations [46]. In this study, we observed significant differences in virus accumulation among cultivars carrying the same resistance genes. The exact cause of this discrepancy remains unclear. One possibility is that these cultivars, despite sharing resistance genes (*Wsm1*, *Wsm2*, *Cmc3*, or *Cmc4*), differ in their genetic backgrounds, which may modulate virus accumulation. As noted earlier, this variation could also influence the virus titers in mites feeding on these cultivars.

## 5. Conclusions

Current WSM management in the Great Plains depends mainly on planting cultivars resistant to either WSMV or WCM. This work shows that TriMV is a key driver of mixed infection which leads to a more severe WSM disease phenotype. This increase in TriMV accumulation in mixed infected plants translates into TriMV accumulation in mites which plausibly leads to the increased transmission of TriMV by mites. This partly explains the increasing incidence of TriMV across the Great Plains. This one-of-a-kind study documented the adverse impacts of individual or mixed infection on mite fecundity, especially in *Wsm1*- and *Wsm2*- resistant plants. Furthermore, virus accumulation in mites was density-dependent, in that higher virus titers in mites were consistent with those in the plants on which they fed. This is likely to have consequences in the field as differential inoculation could lead to the differential spread of the disease. *Cmc3* and *Cmc4* appeared to provide protection that was relatively more complete against this complex pathosystem. Understanding the pathogen dynamics and transmission in the field is critical for deepening our understanding of this complex pathosystem and for devising effective WSM management strategies. More efforts are warranted to characterize the current sources of resistance, identify new resistances, and incorporate them into elite wheat cultivars.

## Figures and Tables

**Table 1:** Wheat cultivars used in the experiments.

Sr. No.	Cultivar	Trait	Resistant Gene
1	Mace	Resistant to WSMV ^a^ and TriMV ^b^	*Wsm1*
2	Breakthrough		
3	RonL	Resistant to WSMV	*Wsm2*
4	Guardian		
5	Joe		
6	TAM 205		
7	TAM 112	Resistant to WCM ^c^	*Cmc3* and *Cmc4*
8	TAM 115		*Cmc 4*
9	TAM 204		
10	TAM 304	Susceptible	None

^a^ Wheat streak mosaic virus; ^b^ Triticum mosaic virus; ^c^ Wheat curl mite.

**Table 2:** Results of mite-mediated TriMV and/or WSMV transmission experiments based on PCR detection of TriMV and/or WSMV RNA in inoculated plants corresponding to different wheat cultivars.

		Cultivars Carrying *Wsm1* Gene								Cultivars Carrying *Wsm2* Gene																Cultivars Carrying *Cmc3* and *Cmc4*				Cultivars Carrying *Cmc4*								Susceptible			
		Mace				Breakthrough				Guardian				RoNL				Joe				TAM 205				TAM 112				TAM 115				TAM 204				TAM 304			
		T	W	T + W	M − I	T	W	T + W	M − I	T	W	T + W	M − I	T	W	T + W	M − I	T	W	T + W	M − I	T	W	T + W	M − I	T	W	T + W	M − I	T	W	T + W	M − I	T	W	T + W	M − I	T	W	T + W	M − I
% infection		70	55	75	0	70	55	75	0	60	65	70	0	50	80	65	0	75	75	75	0	70	85	90	0	70	65	55	0	70	50	70	0	60	55	70	0	75	90	90	0

^T^ Triticum mosaic virus-infected plants inoculated by mite feeding of wheat plants infected with Triticum mosaic virus. ^W^ Wheat streak mosaic virus-infected plants inoculated by mite feeding of wheat plants infected with wheat streak mosaic virus. ^T + W^ Mixed infected plants inoculated by mite feeding of wheat plants infected with Triticum mosaic virus and wheat streak mosaic virus. ^M − I^ Mock-inoculated plants with non-viruliferous mites feeding on non-infected wheat plants.

## Data Availability

The raw data from this study will be made available upon request to the corresponding author.
